# Chamaejasmine Induces Apoptosis in Human Lung Adenocarcinoma A549 Cells through a Ros-Mediated Mitochondrial Pathway

**DOI:** 10.3390/molecules16108165

**Published:** 2011-09-27

**Authors:** Hongyang Yu, Tingting Zhang, Li Cai, Yuanyuan Qu, Songliu Hu, Guanglu Dong, Rongwei Guan, Xiangying Xu, Lina Xing

**Affiliations:** 1 Department of Radiation Oncology, The Second Affiliated Hospital, Harbin Medical University, Harbin 150086, China; 2 Department of Radiation Oncology, The Third Affiliated (Tumor) Hospital, Harbin Medical University, Harbin 150040, China; 3 Oncology Department of Internal Medicine, The Third Affiliated (Tumor) Hospital, Harbin Medical University, Harbin 150040, China; 4 Harbin Medical University Cancer Research Institute, Harbin 150040, China; 5 Key Laboratory of Medical Genetics (Harbin Medical University), Heilongjiang Higher Education Institutions, Harbin 150081, China

**Keywords:** chamaejasmine, A549, Apoptosis, ROS, mitochondrial

## Abstract

In the present study, the anticancer activity of chamaejasmine towards A549 human lung adenocarcinoma cells was investigated. In order to explore the underlying mechanism of cell growth inhibition of chamaejasmine, cell cycle distribution, ROS generation, mitochondrial membrane potential (Δψ_m_) disruption, and expression of cytochrome c, Bax, Bcl-2, caspase-3, caspase-9 and PARP were measured in A549 cells. Chamaejasmine inhibited the growth of A549 cells in a time and dose-dependent manner. The IC_50_ value was 7.72 µM after 72 h treatment. Chamaejasmine arrested the cell cycle in the G2/M phase and induced apoptosis via a ROS-mediated mitochondria-dependent pathway. Western blot analysis showed that chamaejasmine inhibited Bcl-2 expression and induced Bax expression to desintegrate the outer mitochondrial membrane and causing cytochrome c release. Mitochondrial cytochrome c release was associated with the activation of caspase-9 and caspase-3 cascade, and active-caspase-3 was involved in PARP cleavage. All of these signal transduction pathways are involved in initiating apoptosis. To the best of our knowledge, this is the first report demonstrating the cytotoxic activity of chamaejasmine towards A549 *in vitro*.

## 1. Introduction

Lung cancer is one of the most common causes of cancer-related deaths all over the world [1]. Either cisplatin or platinum-based chemotherapy is the most common regimen for treating patients with advanced lung cancer. However, these drugs (especially cisplatin) are highly toxic with a low survival profile against non-small cell lung cancer (NSCLC) [2,3]. Thus, an understanding of the molecular mechanisms of more effective and less harmful therapies are needed to reduce lung cancer mortality. 

Apoptosis is a form of programmed cell death. It occurs through activation of the cell-intrinsic suicide machinery and is a hallmark of action of many anticancer drugs [4,5,6,7]. It is characterized by particular morphological changes, including plasmamembrane bleb, cell shrinkage, depolarization of mitochondria, chromatin condensation, and DNA fragmentation [8]. Activation of the apoptotic cascade results from a complex interaction of molecular events [9]. ROS generation and disruption of the mitochondrial membrane potential contributed to drugs-induced apoptosis [10,11]. The mitochondria-dependent pathway for apoptosis is governed by Bcl-2-family proteins [12]. Bax/Bcl-2 regulates the release of cytochrome c from mitochondria into the cytosol, and the cytochrome c in the cytosol initiates caspases cascade which terminates cells to apoptosis [13]. The relationship between apoptosis and cancer has been closely studied recently. Apoptosis provides a number of useful clues when generating effective therapies and many chemotherapeutic agents exert their anticancer effects by inducing apoptosis in cancer cells [14]. Therefore, induction of apoptosis has become an important mechanism by which anticancer therapy is effective [15].

**Figure 1:**
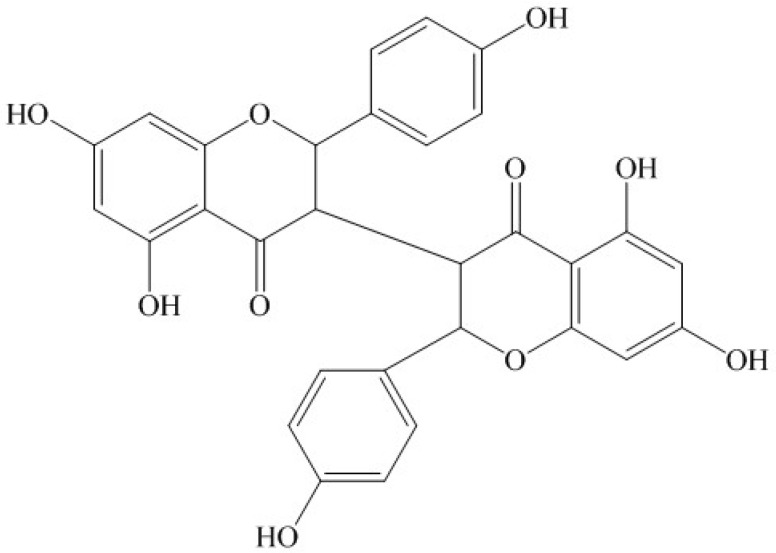
Chemical structure of chamaejasmine.

Many medical plants have served as anticancer pharmaceutical resources, and over 60% of current anticancer drugs such as vinblastine, topotecan, etoposide, and paclitaxel were originally plant-derived compounds [16,17]. *Stellera chamaejasme* L. (Thymealaeaceae) is widely distributed in Northwest and Southwest China. The roots of *S. chamaejasme* L., can be used as pesticide on bugs, flies and maggots, and can also control pests on crops and pastures [18,19]. It has also been found that the methanol extracts of the root of *S. chamaejasme* L. showed significant antitumor activities [20]. Chemical constituent investigations indicated *S. chamaejasme* L. is rich in biflavonones which have been considered as being responsible for the beneficial effects of *S. chamaejasme* L. on human health [21]. Chamaejasmine (Figure 1) is a natural biflavanone with notable pesticidal activity [22]. However, the anticancer activity of chamaejasmine has not been elucidated yet. In the present study, we first examined the growth-inhibitory effect of chamaejasmine on human lung adenocarcinoma A549 cells by the MTT (3-(4,5)-dimethylthiazoly1)-3,5-diphenytetrazolium bromide) assay. Cell cycle, apoptosis analysis, generation of ROS, and mitochondrial membrane potential were studied by flow cytometry. The expression of cytochrome c, PARP, Bax, Bcl-2, caspase-3 and caspase-9 proteins was further assayed by Western blotting to assess apoptosis.

## 2. Results

### 2.1. Cytotoxicity Assays

**Figure 2:**
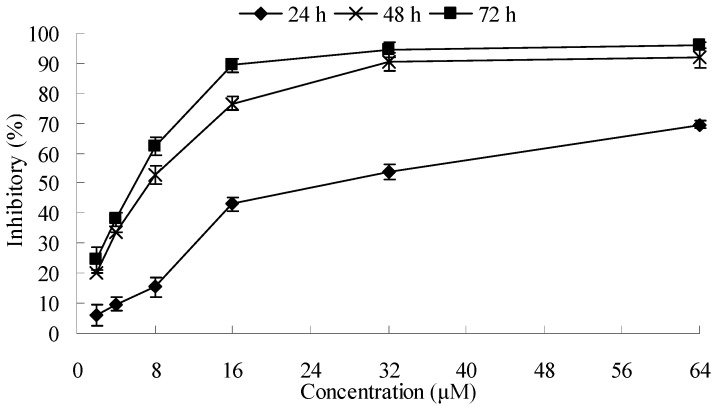
Effect of chamaejasmine towards A549 cells as determined by the MTT assay. The values for each chamaejasmine concentration tested represent the average (mean ± S.D.) from eight replicate wells and are representative of three separate experiments.

The cytotoxicity of chamaejasmine was evaluated on four human cancer cell lines (A549, H1975, SMMC-7721 and SKOV-3) by the MTT assay. Taxol was used as positive control and the results are listed in Table 1. As shown in the Table, chamaejasmine showed more notable cytotoxicity against A549 than H1975, SMMC-7721 and SKOV-3, with IC_50_ values of 7.72, 18.11, 14.04 and 10.43 µM, respectively. Taxol showed even stronger inhibition against all tested cell lines compared with chamaejasmine, with IC_50_ values ranging from 3.98 to 12.39 (*p* < 0.05). As shown in Figure 2, chamaejasmine also reduced proliferation of A549 cells in a time-dependent manner.

### 2.2. Flow Cytometric Analysis of A549 Cell Cycle Distribution and Apoptosis

**Figure 3:**
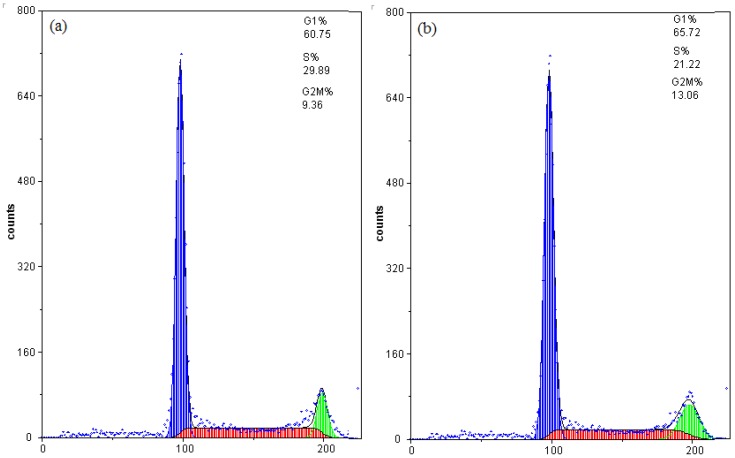

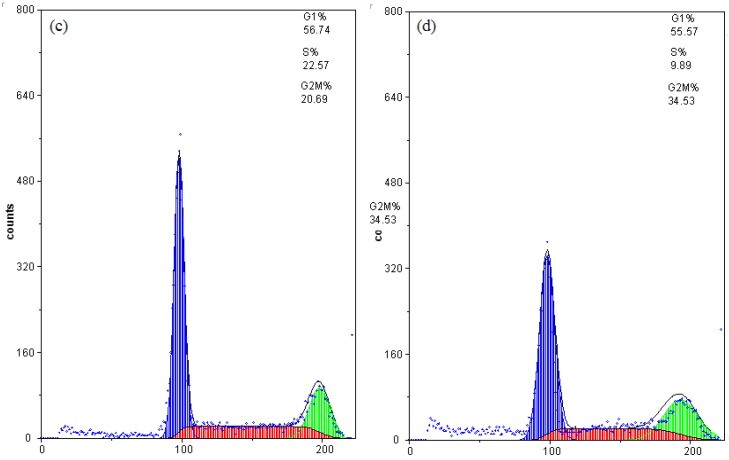
Cell cycle distribution of A549 cells after treatment with different concentrations of chamaejasmine for 48 h. (**a**) treatment with 0 µM chamaejasmine; (**b**) treatment with 2 µM chamaejasmine; (**c**) treatment with 4 µM chamaejasmine; (**d**) treatment with 8 µM chamaejasmine. Blue = G1; red = S; green = G2/M.

To determine whether chamaejasmine-induced apoptosis was related to arrest of cell cycle progression in A549 cells, flow cytometry was used to quantitate the cell cycle distribution under treatment with different chamaejasmine concentrations (2–8 µM). As shown in the concentration kinetic measurements (Figure 3), exposure to 2–8 µM chamaejasmine caused an increase of the G2/M phase population from 13.06% to 34.53%, as compared to 9.36% of G2/M phase cells in untreated control samples. Hence, chamaejasmine exerted growth-inhibitory effects via G2/M phase arrest in a concentration-dependent manner.

**Figure 4:**
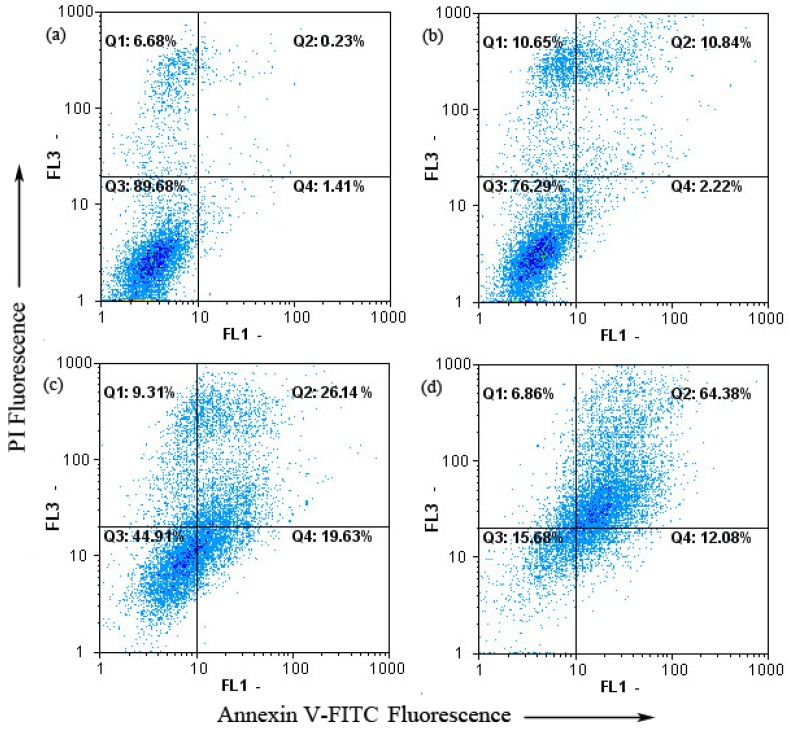
Chamaejasmine-induced apoptosis in A549 cells using annexinV-FITC/PI. (**a**)-(**d**) Treatment with 0, 2, 4 and 8 µM chamaejasmine, respectively.

The Annexin V-FITC apoptosis detection kit was then used to examine the influence of chamaejasmine on A549 cell apoptosis by flow cytometry. As shown in Figure 4, only a small percentage of untreated A549 (1.64%) cells bound with annexinV-FITC. In contrast, the percentage of annexinV-FITC binding A549 cells significantly increased in a concentration-dependent manner after treatment with 2–8 µM chamaejasmine (13.06% to 76.46%, *p* < 0.05). To summarize, data points were dispersed and shifted to the Q2 side in a dose-dependent manner when A549 cells were treated with chamaejasmine, indicating that the cells moved to the late apoptotic stage. These experimental results demonstrate that chamaejasmine induced apoptosis of A549 cells.

### 2.3. Measurement of Reactive Oxygen Species (ROS)

**Figure 5:**
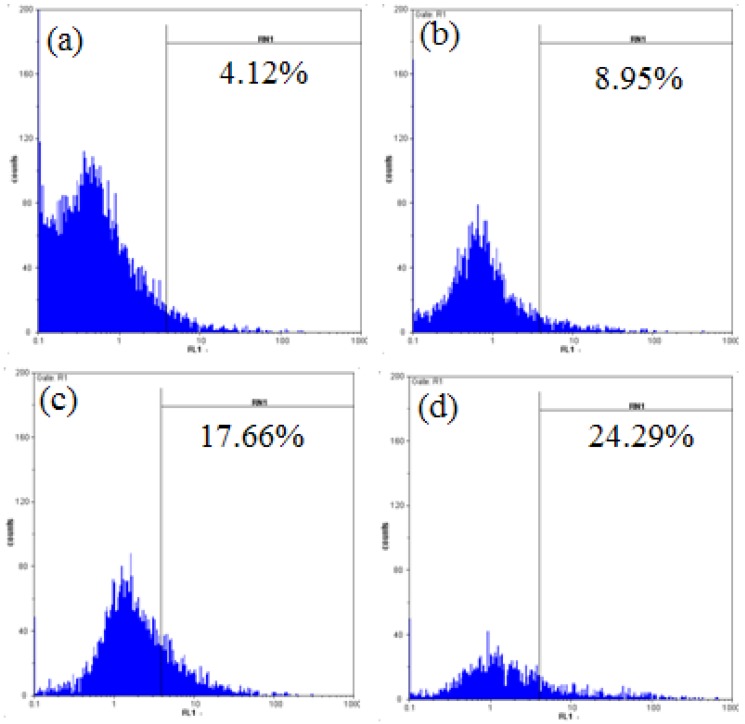
Effect of chamaejasmine on intracellular ROS formation in A549 cells. (**a**)-(**d**) Treatment with 0, 2, 4 and 8 µM chamaejasmine, respectively.

Generation of ROS of A549 induced by chamaejasmine treatment was measured by DCFH-DA and flow cytometry as an indicator of peroxides and superoxide accumulation. Upon challenge of A549 cells for 48 h with chamaejasmine, a concentration-dependent increase of ROS production was observed (Figure 5). Mean fluorescence intensity of untreated cells was 4.12%, and the values changed to 8.95%, 17.66% and 24.29%, after treatment with 2, 4 and 8 µM chamaejasmine, respectively. Fluorescence intensities of chamaejasmine-treated A549 cells were much higher than those of untreated controls (*p* < 0.05).

### 2.4. Disruption of Δψm and Cytochrome C Release during Chamaejasmine Induced Apoptosis 

**Figure 6:**
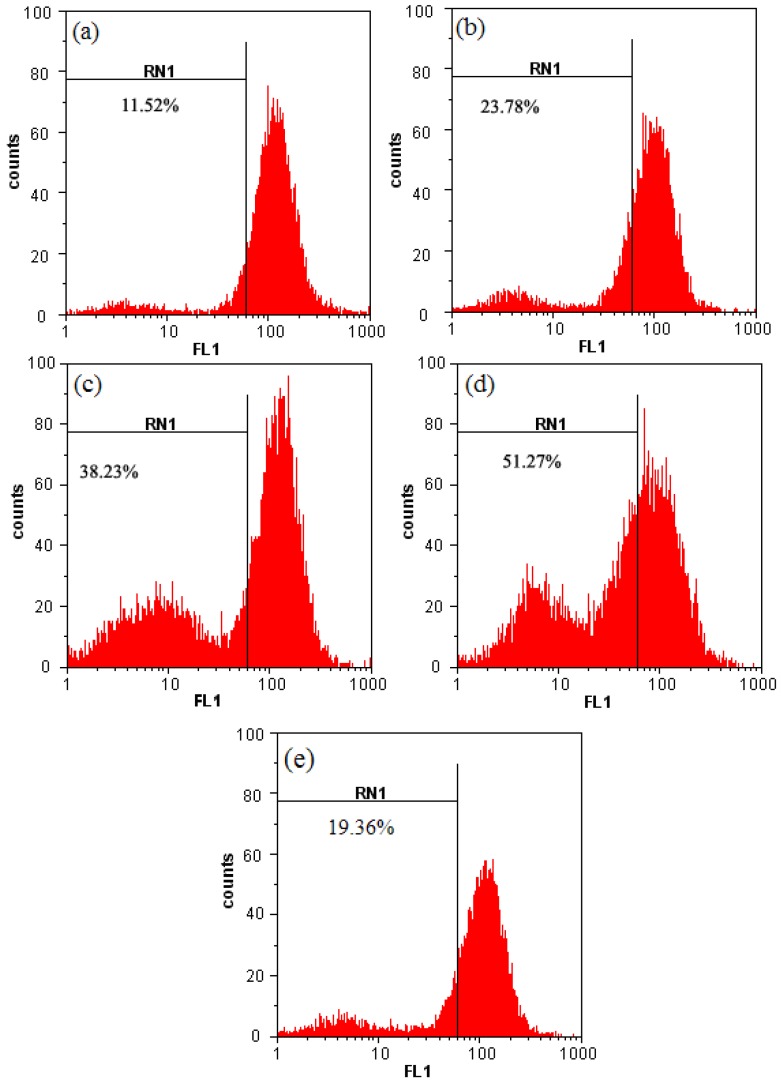
Mitochondrial membrane potential of A549 cells after treatment with chamaejasmine using Rh123 staining. (**a**)-(**d**) Treatment with 0, 2, 4 and 8 µM chamaejasmine, respectively; (**e**). Treatment with 5 mM NAC and 8 µM chamaejasmine; These figures are one representative experiment of three with similar results.

To assess the effects of chamaejasmine on the mitochondrial apoptotic pathway, the Δψ_m_ in A549 cells treated with different concentrations of chamaejasmine was measured using rhodamine 123 as fluorescent dye. As shown in Figure 6, the number of cells with depolarized mitochondria increased with chamaejasmine dose. The addition of chamaejasmine at doses of 0–8 µM led to increasing percentages of Δψ_m_ disruption from 11.52% to 51.27% (*p* < 0.05). To establish a link between the mitochondrial events and the oxidative stress, we tried to inhibit the intracellular alteration of redox state using N-acetylcysteine (NAC), a well-known reactive oxygen species scavenger [23]. ΔΨm loss reached their maximum after treated with 8 µM chamaejasmine, the point therefore was used to measure the effect of NAC on ΔΨm. As shown in Figure 6(e), the reduction of ΔΨm in chamaejasmine-treated A549 cells was reduced obviously in the presence of 5 mM NAC. This result suggests that reactive oxygen species may be a requirement for chamaejasmine mediated mitochondria alterations in A549 cells.

**Figure 7:**
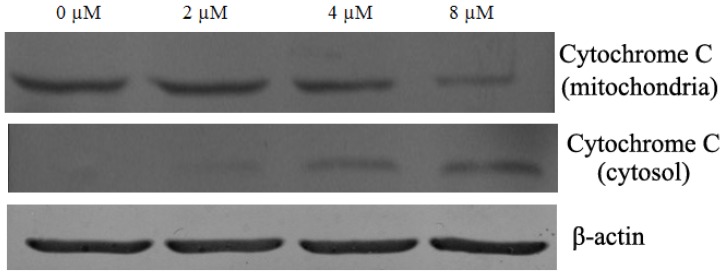
Chamaejasmine -induce cytochrome c release by western blotting assay, A549 cells were treated with chamaejasmine (0, 2, 4, 8 µM) for 48 h, respectively. These figures are one representative experiment of three with similar results. β-actin served as a loading control.

During activation of the mitochondrial-dependent apoptotic pathway, a number of signals can cause activation of the mitochondrial permeability transition and concomitant release of cytochrome c. Cytochrome c was greatly increased in the cytosol of cells treated with chamaejasmine which indicating cytochrome c release from mitochondria to cytoplasm (Figure 7). These results confirmed the involvement of mitochondria in chamaejasmine-induced apoptosis.

### 2.5. Chamaejasmine-Mediated Up-Regulation of Bax and Down-Regulation of Bcl-2

**Figure 8:**
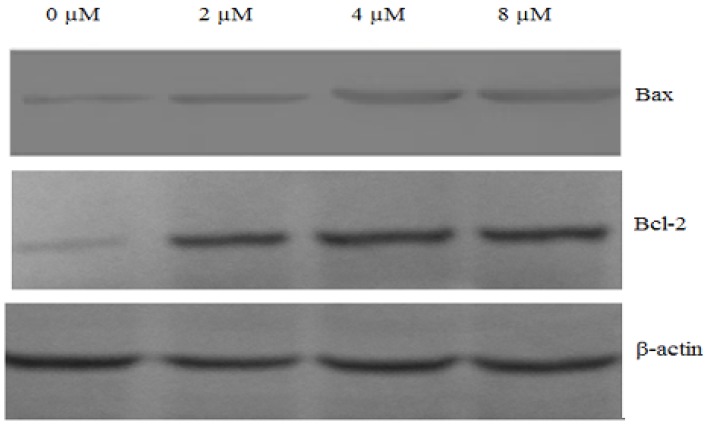
Chamaejasmine-mediated up-regulation of Bax and downregulation of Bcl-2 by western blotting assay, A549 cells were treated with chamaejasmine (0, 2, 4, 8 µM) for 48 h, respectively. The test was repeated three times and representative blots are shown. β-actin served as a loading control.

The expression of the pro-apoptotic protein Bax and the anti-apoptotic protein Bcl-2 are crucial determinants of the apoptotic response mediated by many agents. A high Bax/Bcl-2 ratio was clearly correlated with increased apoptotic sensitivity to anti-cancer reagents [24]. Western blot analysis revealed a significant increase in the expression of Bax in chamaejasmine treated cells, while there was a significant decrease in Bcl-2 expression (Figure 8), indicating that the Bax/Bcl-2 ratio increased significantly.

### 2.6. Chamaejasmine Induces Activation of Caspase-3, -9 and PARP

**Figure 9:**
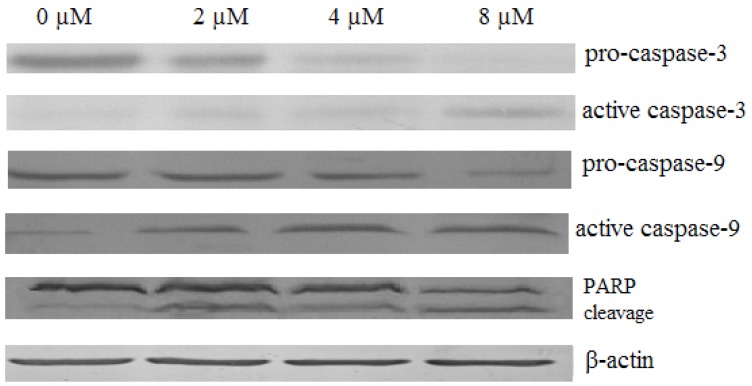
Effects of chamaejasmine on expression of caspases and PARP. A549 cells were treated with different concentrations of chamaejasmine for 48 h. The full length and cleaved protein fragments of caspase-3, caspase-9, and PARP were detected by Western blot. The results demonstrated dose-dependent chamaejasmine induced cleavage of caspase-3, caspase-9 and PARP. These figures are one representative experiment of three with similar results. β-actin served as a loading control.

To study the possible mechanism of chamaejasmine-induced apoptosis on A549 cells, we investigated the activation of caspases and PAPR. After 48 h exposure to chamaejasmine, it was found that pro-caspase-9, pro-caspase-3, and pro-PARP were cleaved to their active forms and the level of active protein increased with chamaejasmine dose (Figure 9).

## 3. Discussion

This study is the first to examine the effect of chamaejasmine on anti-proliferative activity toward human lung adenocarcinoma A549 cells. Our experimental results showed that chamaejasmine has obvious cytotoxicity against A549 cells, based on the significant IC_50_ value of 7.72 µM (Table 1). Many biflavones possess anti-tumor activity towards various human cancer cell lines, suggesting that they may be promising candidates for novel anticancer agents [25]. Amentoflavone obtained from *Selaginella tamariscina* showed certain anticancer activity against Hela and MCF-7, with IC_50_ values of 76.83 µM, 67.71 µM, respectively. It has potential for cancer chemoprevention [25]. Chamaejasmine has a similar chemical structure, but showed lower IC_50_ values in the present investigation. Therefore, we have reason to believe that the potential of chamaejasmine in cancer therapy is more promising than that of amentoflavone.

Cell cycle control is a major event in cellular division. Recently, blockade of the cell cycle is considered as an effective strategy for the development of novel cancer therapies [26,27]. In the present study, cell cycle analysis of the treated culture revealed that chamaejasmine induced a concentration-dependent G2/M phase cell cycle arrest with an accompaniment decrease in G1 and S phase. This result confirmed that chamaejasmine inhibited DNA synthesis and induced a block at the G2/M boundary. The cell cycle arrest may partly explain apoptosis and anti-proliferative effects induced by chamaejasmine.

Apoptosis plays an important role in anticancer effect. It is a highly regulated death process by which cells undergo inducible non-necrotic cellular suicide [28]. Data obtained from flow cytometric annexinV-FITC/PI staining showed that chamaejasmine induced apoptosis in A549 cells. Therefore, we further evaluated the changes of ROS and Δψ_m_ in A549 cells as well as the expression of apoptosis related proteins. Our results showed that chamaejasmine generated ROS in a dose-dependent manner in A549 cells. Increased levels of ROS are commonly known to cause mitochondrial membrane depolarization [29]. Accordingly, we also found Δψ_m_ decreased in chamaejasmine-treated A549 cells. Since mitochondrial membrane depolarization is one of the earliest intracellular events of apoptosis [30,31], we could conclude that ROS generation by chamaejasmine was responsible for disruption of the mitochondrial membrane potential. Results suggested that intracellular ROS plays an important role in chamaejasmine-induced apoptosis.

Bcl-2 family proteins play important roles in apoptosis regulation. Anti-apoptotic (e.g. Bcl-2 and Bcl-xL) and pro-apoptotic (e.g., Bad, Bax and Bak) are two of the major members in Bcl-2 family [32,33,34]. Anti-apoptotic Bcl-2 and Bcl-xL inhibit apoptosis by sequestering proforms of capsases or by preventing the release of mitochondrial apoptogenic factors [35,36], whereas, bad, Bax and Bak inhibit Bcl-2 activity and promote apoptosis [37]. In this study, chamaejasmine treatments altered the expression of anti-apoptotic (Bcl-2) and pro-apoptotic (Bax) proteins, resulting in A549 cells apoptosis (Figure 8). Moreover, decreased mitochondrial membrane potential regulates mitochondrial permeability transition pore (MPT) opening [38] which is associated with cytochrome c release [39]. High Bax/Bcl-2 ratio also resulted in cytochrome c release [40]. Chamaejasmine decreased Δψ_m_ and increased Bax/Bcl-2 ratio, both of which could explain chamaejasmine-induce cytochrome c release.

**Figure 10:**
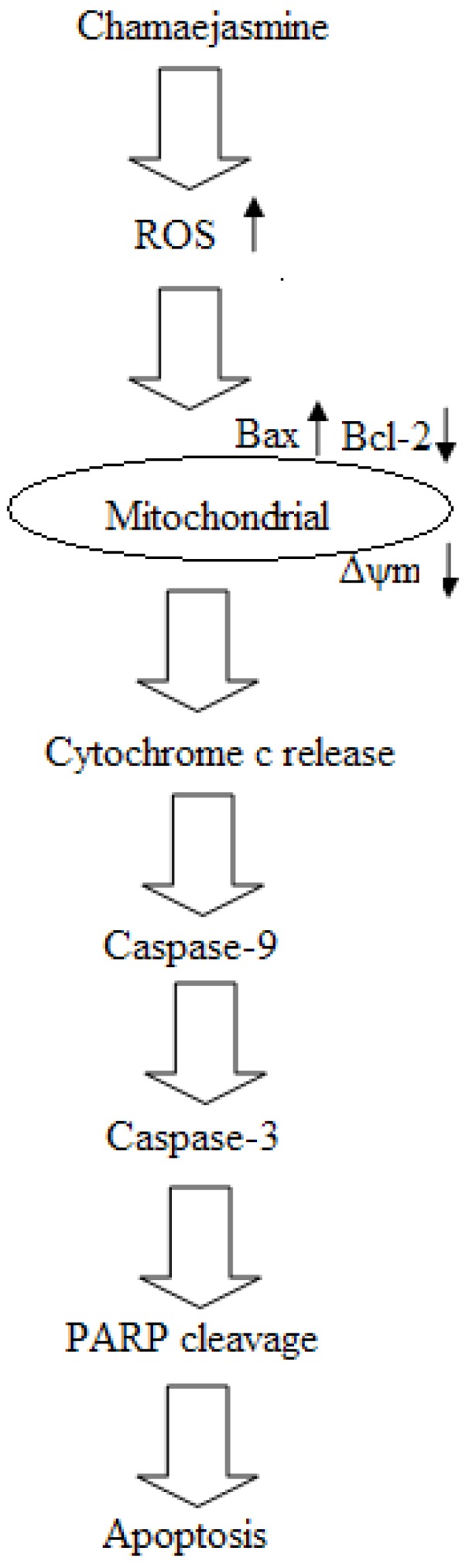
Signaling pathway of chamaejasmine-induced apoptosis in A549 cells.

Cytochrome c binds with Apaf-1 and procaspase-9 in a dATP-dependent manner to form the apoptosome [41]. The apoptosome can induce activation of caspase-9 which activates the effector pro-caspases, including pro-caspase-3, an effector caspase of apoptosis [42]. Caspase-3 is a well-known downstream adaptor caspase which can be proteolytically activated by caspase-9 via mitochondrial or cell death receptor signaling pathways [43,44]. PARP represents an intrinsic substrate for caspase-3 [45] and is cleaved upon caspase-3 activation. PARP is a highly conserved nuclear enzyme tightly binding to DNA with importance for DNA repair, recombination, proliferation and genomic stability [46]. Cleavage of PARP is an early and critical event required for tumour cells apoptosis [47]. The activated caspase-9, caspase-3 and the cleavage of PARP detected in the results further explained clearly the signaling pathway of chamaejasmine-induced apoptosis in A549 cells (Figure 10). Further studies on the *in vivo* activity of chamaejasmine towards A549 xenograft tumors in nude mice are eagerly needed.

## 4. Experimental

### 4.1. Cell Growth and Chemicals

The human lung adenocarcinoma (A549) cells, Non-small cell lung carcinoma cell line (H1975), human hepatoma cells (SMMC-7721) and human adenocarcinoma (SKOV-3) were purchased from Harbin Medical University (Harbin, China). Cells were maintained in RPMI 1640 medium supplemented with 10% fetal bovine serum and 100 U/mL penicillin and 100 µg/mL streptomycin. The cells were kept at 37 °C in a humidified atmosphere containing 5% CO_2_. Chamaejasmine and taxol (purity ≥ 99%) were obtained from Sigma-Aldrich Chemical Co. (St. Louis, MO, USA). A 10 mM stock solution of chamaejasmine and taxol was prepared in dimethyl sulfoxide (DMSO) and stored at −80 °C. MTT, rhodamine 123 (Rh123) and propidium iodide (PI) were also obtained from Sigma–Aldrich Inc. N-acetyl-L-cysteine (NAC) was obtained from Calbiochem (San Diego, CA, USA). Deionized water was used in all experiments.

### 4.2. Cytotoxicity Assay

Inhibition of cell proliferation of chamaejasmine was measured by the MTT assay [47]. Briefly, A549, H1975, SMMC-7721 and SKOV-3 cells were plated in 96-well culture plates (1 × 10^5^ cells/well) separately. After 24 h incubation, cells were treated with chamaejasmine (2, 4, 8, 16, 32 and 64 µM, eight wells per concentration) for 24, 48 h; or 72 h, MTT solution (5 mg/mL) was then added to each well. After 4 h incubation, the formazan precipitate was dissolved in dimethyl sulfoxide (100 µL) and then the absorbance was measured in an ELISA reader (Thermo Molecular Devices Co., Union City, USA) at 570 nm. The cell viability ratio was calculated by the following formula: Inhibitory ratio (%) = ((OD_control_ − OD_treated_)/(OD_control_)) × 100%. Cytotoxicity was expressed as the concentration of chamaejasmine inhibiting cell growth by 50% (IC_50_ value).

### 4.3. Flow Cytometric Analysis of Cell Cycle and Apoptosis

Cell cycle was studied with CyStain (Partec GmbH, Görlitz, Germany) [48]. Briefly, 1 × 10^6^ cells/well A549 cells were seeded in six-well plate and left for 24 h in incubator to resume exponential growth. Cells were exposed to chamaejasmine (0, 2, 4 and 8 µM) and incubated for 48 h. Then, the cells were harvested and washed with PBS. After suspension in PBS (800 µL) and CyStain (200 µL) the cell cycle distribution of 10,000 cells was recorded by flow cytometry (Partec), and the percentage of cells at G0/G1, S, and G2/M phases was analyzed using the FloMax software (Partec).

The extent of apoptosis was measured through annexinV-FITC apoptosis detection kit (Beyotime Institute of Biotechnology, Shanghai, China) as described in the manufacturer’s instructions [48]. After exposure to chamaejasmine (0, 2, 4 and 8 µM) for 48 h, cells were collected, washed twice with PBS, gently resuspended in annexinV binding buffer and incubated with annexinV-FITC/PI in dark for 15 min and analyzed by flow cytometry using the FloMax software. The fraction of cell population in different quadrants was analyzed using quadrant statistics. The lower left quadrant contained intact cells; lower right quadrant apoptotic and in the upper right quadrant necrotic or post-apoptotic cells.

### 4.4. Measurement of ROS Generation

ROS generation was analysised by flow cytometry using DCFH-DA [49]. Single-cell suspensions of cells treated with chamaejasmine (0, 2, 4 and 8 µM) for 48 h were prepared in PBS supplemented with 50 mM glucose, and incubated with 10 µM DCFH-DA at 37 °C for 30 min. Fluorescence generation due to the hydrolysis of DCFH-DA to dichlorodihydrofluorescein (DCFH) by non-specific cellular esterases and the subsequent oxidation of DCFH by peroxides was measured by means of flow cytometry.

### 4.5. Changes of Mitochondrial Membrane Potential (Δψm)

The uptake of the cationic fluorescent dye rhodamine 123 has been used for the evaluation of mitochondrial membrane potential [50]. A549 cells were seeded at 1 × 10^6^ cells/well into 6-well plates. After 24 h incubation, cells were treated with serial dilutions of chamaejasmine (0, 2, 4 and 8 µM) for 48 h. Untreated controls and treated cells were harvested and washed twice with PBS. The cell pellets were then resuspended in 2 mL of fresh incubation medium containing 1.0 µM rhodamine 123 and incubated at 37 °C in a thermostatic bath for 30 min with gentle shaking. To further investigate the effects of reactive oxygen species scavenger on chamaejasmine induced apoptosis in A549 cells, cells were pre-incubated for 2 h with 5 mM N-acetyl-L-cysteine (NAC) before the addition of chamaejasmine. A549 cells were separated by centrifugation and washed twice with PBS, then analyzed by means of flow cytometry.

### 4.6. Western Blot Assay

To evaluate the expression levels of various intracellular proteins related to apoptosis, A549 cells were treated with chamaejasmine (0, 2, 4 and 8 µM) for 48 h, respectively. For isolation of total protein fractions, cells were collected, washed twice with ice-cold PBS, and lysed using cell lysis buffer [20 mM Tris pH 7.5, 150 mM NaCl, 1% Triton X-100, 2.5 mM sodium pyrophosphate, 1 mM EDTA, 1% Na_2_CO_3_, 0.5 µg/mL leupeptin, 1 mM phenylmethanesulfonyl fluoride (PMSF)]. The lysates were collected by scraping from the plates and then centrifuged at 10,000 rpm at 4 °C for 5 min. Total protein samples (20 µg) were loaded on a 12% of SDS polyacrylamide gel for electrophoresis, and transferred onto PVDF transfer membranes (Millipore, Billerica, MA, USA) at 0.8 mA/cm^2^ for 2 h. Membranes were blocked at room temperature for 2 h with blocking solution (1% BSA in PBS plus 0.05% Tween-20). Membranes were incubated overnight at 4 °C with primary antibodies (anti-β-actin, anti-Bax, anti-caspase-9, anti-caspase-3 and anti-cytochrome c were mouse polyclonal antibodies; anti-Bcl-2 and anti-PARP were rabbit polyclonal antibodies) at a 1:1000 dilution (Biosynthesis Biotechnology Company, Beijing, China) in blocking solution. After thrice washings in TBST for each 5 min, membranes were incubated for 1 h at room temperature with an alkaline phosphatase peroxidaseconjugated anti-mouse secondary antibody at a dilution of 1:500 in blocking solution. Detection was performed by the BCIP/NBT Alkaline Phosphatase Color Development Kit (Beyotime Institute of Biotechnology) according to the manufacturer’s instructions. Bands were recorded by a digital camera (Nikon, Tokyo, Japan).

### 4.7. Statistical Analysis

The data were expressed as mean ± S.D. All statistics were calculated using the STATISTICA program (StatSoft, Tulsa, OK, USA). A *p*-value of <0.05 was considered as significant.

## 5. Conclusions

In summary, the present study showed that chamaejasmine inhibited the growth of A549 cells in a dose-dependent manner and the reduction in cell viability resulted from cell cycle arrest at G2/M phase, accompanied by cell apoptosis. Chamaejasmine-induced A549 cells apoptosis by a ROS-mediated mitochondria-dependent pathway, involving inhibition of Bcl-2 expression and induction of Bax expression to disintegrate the outer mitochondrial membrane and to cause cytochrome c release. Further downstream of the apoptosis cascade, chamaejasmine activated caspase-9 and caspase-3 leading to PARP cleavage. All these evidences provide a rationale to explore chamaejasmine as a preventive and perhaps as a chemotherapeutic agent in the management of lung cancer.

## Figures and Tables

**Table 1:** Inhibition concentrations 50% (IC_50_) values for chamaejasmine towards A549 cells determined by MTT assay. The symbols * indicate significant differences (*p* < 0.05) with respect to positive control (taxol).

Cell line	IC_50_ (µM)	
	Chamaejasmine	Taxol
A549	7.72 *	4.06
H1975	18.11 *	9.75
SMMC-7721	14.04 *	12.39
SKOV-3	10.43 *	3.98
